# Diminishing-returns epistasis among random beneficial mutations in a multicellular fungus

**DOI:** 10.1098/rspb.2016.1376

**Published:** 2016-08-31

**Authors:** Sijmen Schoustra, Sungmin Hwang, Joachim Krug, J. Arjan G. M. de Visser

**Affiliations:** 1 Laboratory of Genetics, Wageningen University, Wageningen, The Netherlands; 2 Institute of Theoretical Physics, University of Cologne, Cologne, Germany

**Keywords:** Fisher's geometric model, epistasis, *Aspergillus nidulans*, adaptation, beneficial mutation

## Abstract

Adaptive evolution ultimately is fuelled by mutations generating novel genetic variation. Non-additivity of fitness effects of mutations (called epistasis) may affect the dynamics and repeatability of adaptation. However, understanding the importance and implications of epistasis is hampered by the observation of substantial variation in patterns of epistasis across empirical studies. Interestingly, some recent studies report increasingly smaller benefits of beneficial mutations once genotypes become better adapted (called diminishing-returns epistasis) in unicellular microbes and single genes. Here, we use Fisher's geometric model (FGM) to generate analytical predictions about the relationship between the effect size of mutations and the extent of epistasis. We then test these predictions using the multicellular fungus *Aspergillus nidulans* by generating a collection of 108 strains in either a poor or a rich nutrient environment that each carry a beneficial mutation and constructing pairwise combinations using sexual crosses. Our results support the predictions from FGM and indicate negative epistasis among beneficial mutations in both environments, which scale with mutational effect size. Hence, our findings show the importance of diminishing-returns epistasis among beneficial mutations also for a multicellular organism, and suggest that this pattern reflects a generic constraint operating at diverse levels of biological organization.

## 1. Introduction

Adaptive evolution relies on natural selection sorting genetic variation. In clonal populations, beneficial mutations are the only source of genetic variation. In these populations, long-term adaptation is characterized by the fixation of multiple beneficial mutations. Importantly, several studies have shown that the fitness effects of beneficial mutations are not simply additive—a phenomenon called epistasis [1]. Epistasis shapes evolutionary fitness landscapes and affects the dynamics and repeatability of evolution [1–7]. For instance, when a given mutation has a deleterious effect in the presence of a second mutation while having a beneficial effect in the absence of that second mutation, the two mutations are said to display sign epistasis [8]. When sign epistasis is prevalent, adaptation may lead to diverse fitness peaks, whereas few trajectories towards each peak are selectively accessible [1,4,6]. Empirical work has revealed substantial variation in the nature of epistasis across model systems and types of mutations involved [1,9], which prevents understanding the importance and evolutionary implications of epistasis. Interestingly, some recent studies have found support for a model where the beneficial effect of mutations is smaller in the presence of other beneficial mutations and that this negative effect is stronger for larger-benefit mutations than smaller-benefit mutations. This phenomenon is a special case of negative epistasis and also referred to as diminishing-returns epistasis among beneficial mutations [2,10–14]. This pattern suggests a potentially generic organizing principle resulting from global functional (e.g. structural or physiological) constraints and leading to predictive adaptive dynamics, despite variation in the sign and strength of epistasis from local mechanisms [2]. However, studies showing diminishing-returns epistasis are limited in number and scale, and only available for single genes [14], viruses [15,16] and unicellular microbes [2,11–13,17], whereas epistasis is thought to vary with genomic complexity [1,18]. Moreover, available reports of diminishing-returns epistasis are based on co-selected mutations, introducing biases in the estimates of the strength and type of epistasis along the adaptive trajectory [1,19,20]. Further, it has been suggested that a correlation between epistasis and mutation effect size observed in experiments can be artefactual due to the fact that the two terms to be correlated will share measurement errors, which implies a spurious statistical dependence [10].

**Figure 1.**
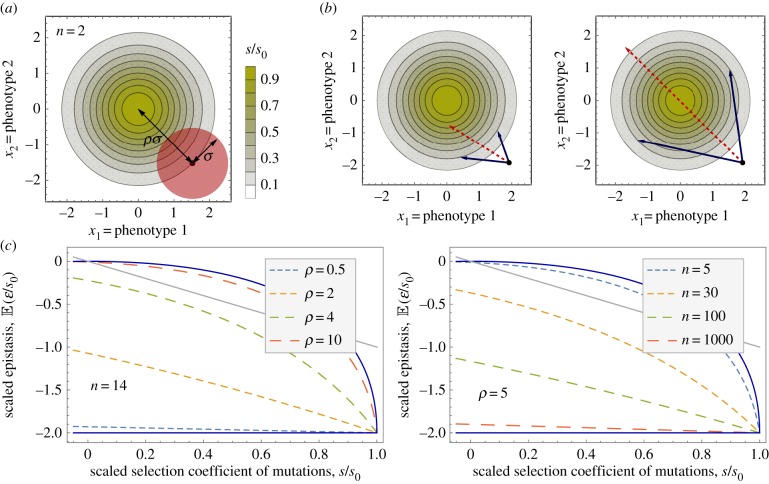
Fisher's geometric model (FGM) and predictions of epistasis among beneficial mutations. (*a*) FGM in two-dimensional phenotype space. The three parameters of FGM include: the distance of the wild-type to the optimum, *ρ* (in terms of the average displacement of mutations, *σ*), the phenotypic dimensions, *n* (here 2), and the fitness difference between the wild-type and the fitness optimum, *s*_0_. (*b*) FGM generates fitness epistasis among beneficial mutations of diverse sign and strength from the nonlinear dependence of fitness on underlying phenotypes. Both diagrams depict effects of single mutations of equal fitness effects (black arrows leading to the same fitness circle). The fitness of the constituent double mutant (red arrows) is nevertheless very different, leading to magnitude epistasis in the left and sign epistasis in the right diagram. (*c*) Predicted relationship between mean epistasis and effect size of beneficial mutations of the same effect-size by FGM for different values of phenotypic dimensions (*n*) and mutational distance to the optimum (*ρ*). The two solid lines are obtained from the limiting behaviours of the mean epistasis obtained by taking the small-*ρ* and large-*ρ* limits, i.e. −2 and 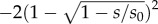, respectively. The straight grey lines show the relationship *ɛ* = −*s*.

Here, we test for diminishing-returns epistasis between independent beneficial mutations in the multicellular fungus *Aspergillus nidulans*. We combine predictions from Fisher's geometric model (FGM) [21], a heuristic phenotype-fitness model with proven utility for describing fitness effects of mutations and their epistatic interactions [16,22–25], with large-scale experimental measurements of epistasis among beneficial mutations. FGM assumes the presence of a single fitness optimum in multidimensional phenotype space, and has as parameters the number of phenotypic dimensions (*n*), the fitness difference between the wild-type and the fitness optimum (*s*_0_), and the average number of beneficial mutations required to reach the optimum (*ρ*; figure 1*a*). The model assumes that each mutation affects multiple phenotypes (called ‘universal pleiotropy’) and multiple mutations act additively per phenotype. Epistasis at the level of fitness then results only from the nonlinear mapping of phenotypes onto fitness [26]. Crucially, FGM predicts a pattern of diminishing-returns epistasis among beneficial mutations, whose shape depends on its parameters [13,27].

## 2. Results and discussion

We use FGM to formulate specific predictions on the shape of the relationship between effect size of beneficial mutations, *s*, and pairwise epistasis between mutations, *ɛ*. We do so for pairs of individually beneficial mutations of similar effect, for which FGM predicts substantial variation in epistatic strength. As illustrated in figure 1*b* for the case of two phenotypic dimensions (*n* *=* 2), the beneficial mutations are represented by two vectors pointing from the wild-type phenotype to a circle comprising all phenotypes at a certain (smaller) distance from the fitness optimum—the fitness optimum being the middle of the circle. The vectors are chosen at random from an isotropic, multivariate Gaussian distribution*.* Depending on where the vectors representing effects of single mutations reach the circle, the selective effect of the double mutant that combines the effect of two single mutations (represented by the dashed arrow) varies widely. This means that by its very construction, FGM introduces a distribution of epistatic effects for a given single effect size *s*, which is characterized by its mean and variance. Within this setting, the variability of the experimental epistasis measurements is therefore interpreted as a combination of the inherent stochasticity of FGM and the external noise induced by measurement errors.

In our experiments, fitness is measured as linear growth rate, and effects of single mutations are expressed in terms of increases in growth rate. Epistasis is defined as deviation from the additive effects of the two single mutations as follows [28]: *ɛ* = Δ*f_ab_* − (Δ*f_a_* + Δ*f_b_*), where Δ*f_a_* and Δ*f_b_* are the fitness effects of the single mutants and Δ*f_ab_* is the fitness effect of the double mutant. For pairs of mutations of identical effect, Δ*f_a_* = Δ*f_b_* = *s*, epistasis is *ɛ* = Δ*f_ab_* − 2*s*, and sign epistasis is distinguished by the condition Δ*f_ab_* < *s* or *ɛ* < −*s*. Analytical results derived in the supplement yield two main predictions (figure 1*c*). First, mean epistasis is negative irrespective of model parameters and the fitness effect of the mutations. Second, mean epistasis shows a diminishing-returns relationship with mutation effect-size that is conspicuously nonlinear for relatively small values of *n* and large values of *ρ*. In addition, the strength of negative epistasis increases for genotypes closer to the optimum (small *ρ*) and with increasing phenotypic dimension (large *n*), in line with previous numerical results [27]. Note that the model predicts negative epistasis even for neutral mutations, although the strength of epistasis is small unless *ρ* is small and *n* is large. An explicit analytical expression for the variance of the epistatic effects is also available, but the full distribution cannot be obtained. Nevertheless, comparison with simulations shows that it is well approximated by a Gaussian (electronic supplementary material, figure S1). The probability for a pair of mutations to display sign epistasis can then be obtained by integrating the distribution up to *ɛ* = −*s*, which shows that sign epistasis is most prevalent for mutations of weak and strong effects but less pronounced at intermediate values of *s* (electronic supplementary material, figure S1).

**Figure 2.**
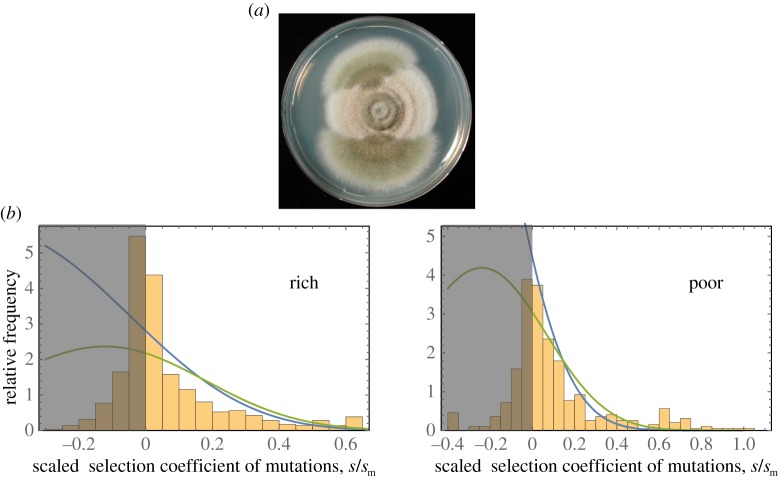
Beneficial mutations in the fungus *Aspergillus nidulans*. (*a*) *A. nidulans* colony growing under suboptimal conditions, producing mutant sectors with higher growth rates. (*b*) Frequency distribution of the relative selection coefficient (i.e. the difference in radial colony growth rate of isolate and ancestor normalized by the maximally fit isolate, *s/s*_m_) of 614 isolates from the edge of colonies growing in a rich nutrient environment (left) and 824 isolates from the edge of colonies growing on a poor nutrient environment (right). The blue lines show the probability density function for fitness effects of beneficial mutations (approximately all data outside the shaded areas) predicted by FGM parametrized by the epistasis data; the green lines show this function parametrized by the distribution of single mutation effects (see electronic supplementary material and figure S4).

To test the analytical predictions, we measured epistasis between independently selected beneficial mutations (i.e. mutations whose interactions have not been affected by natural selection) in the filamentous fungus *A. nidulans*. This species has multicellular mycelium containing haploid nuclei and produces both asexual and sexual spores [28,29]. By growing colonies on solid medium under suboptimal conditions, faster-growing beneficial mutants can readily be detected (figure 2*a*). To (potentially) vary the epistatic properties of isolated beneficial mutations, we grew 614 colonies on rich medium and 824 colonies on poor medium, and collected mycelium samples most distant from the point of inoculation to isolate spores potentially carrying a beneficial mutation. Figure 2*b* shows the distribution of the relative fitness effects of these mutants, where fitness effects are expressed relative to those of the largest-effect mutation in each environment, i.e. the fitness effect of genotype *i* is defined as (*f_i_* − *f*_0_)/*f*_max_, where *f_i_, f*_0_ and *f*_max_ are the growth rates of genotype *i*, the ancestral strain and the strain with highest fitness, respectively. The relative selection coefficient (*s/s*_m_) of a given mutation is the difference in growth rate of that mutant and the ancestor normalized by the highest observed increase (*s*_m_). We purified potential mutants and assayed their fitness by measuring the rate of radial colony growth. A total of 244 isolates (154 on rich and 90 on poor medium) had fitness significantly higher than the ancestor (i.e. outside the 99.99% CI for the fitness of the ancestor) and hence probably carried a single beneficial mutation. The wide range of mutational effects combined with recent genomic data is suggestive of a wide range of mutational targets that result in increased fitness (see Methods; Dettman *et al.* 2016, unpublished data). In figure 2*b*, the empirical distribution of fitness effects is compared with the prediction of FGM using two different parametrizations, derived from single effect sizes and pairwise epistasis, respectively.

**Figure 3.**
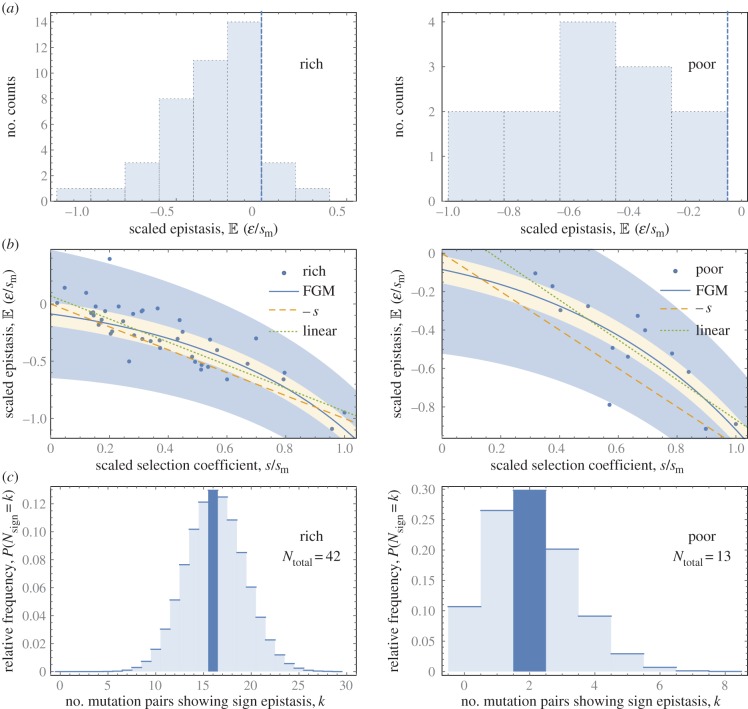
Epistasis among beneficial mutations. (*a*) Histograms of the epistasis coefficients calculated for pairs of beneficial mutations of similar effect isolated in a rich (left) and poor environment (right). (*b*) Epistasis as a function of the mean relative selection coefficient for the two mutations used to generate the double mutant for the rich (left) and poor environment (right). The blue line in each panel is the best-fitted FGM model with parameter values (*s*_0_/*s*_m_ = 1.41, *ρ* = 6.89, *n* = 19.3) and (*s*_0_/*s*_m_ = 1.62, *ρ* = 9.81, *n* = 34.8), respectively. The orange shading indicates the 99% CI based on measurement error, whereas the blue shading indicates the 99% variability region resulting from the combined effect of measurement error and the intrinsic stochasticity of FGM. The orange dashed line in each panel indicates the threshold between magnitude (above the line) and sign epistasis (below the line); the green dashed line is the best-fit linear model. (*c*) Probability density plot of the number of instances of sign epistasis in the rich (left) and poor environment (right) predicted by FGM. Highlighted bars indicate observed numbers of sign epistatic pairs out of the total number of pairs *N*_total_ (see text).

To determine the sign and strength of epistasis between these beneficial mutations, we performed sexual crosses to generate pairwise combinations of mutations of similar fitness effect, using each mutation in one combination only (see Methods). This resulted in a total of 55 successful crosses (42 for rich medium and 13 for poor medium). Fitness effects of the single mutants are measured as differences in linear growth rate of mutant and ancestral strain. Fitness of the double mutant was determined by measuring the growth rate of colonies started with sexual spore samples containing all four recombinant genotypes of each cross (see Methods). Because the colony growth rate of the mixture will generally be determined by the fastest-growing genotype present, our method can only detect magnitude epistasis, and hence yields an upwardly biased estimate of mean epistasis. Note, however, that the data displayed in figure 3*b*, nevertheless, show at least one estimate in both environments significantly below the line –*s,* indicating that sign epistasis can be detected in certain cases. This is presumably owing to a trade-off between growth rate and spore germination, which allows the double mutant to block faster-growing genotypes and determine the rate of colony expansion (see Methods §4e). Such effects have been observed previously in experiments with this species [30].

Our upwardly biased estimates support the first two predictions from FGM. First, mean epistasis is negative for beneficial mutations in both the rich (*t* = −6.92, d.f. = 41, *p* < 0.001) and poor environment (*t* = −6.75, d.f. = 12, *p* < 0.001); only three of the 42 rich environment and none of the 13 poor environment mutation pairs show positive epistasis (figure 3*a*). Mean epistasis is stronger in the poor (−19.25 mm per 5 days) than in the rich environment (−14.94 mm per 5 days), but this difference is not significant (*t* = 1.03, d.f. = 53, *p* = 0.15). Second, the dependence of epistasis on mutation effect size predicted by FGM is evident in the data when tested with a linear model (figure 3*b*; rich environment: *F*_1,41_ = 104, *p* < 0.001; poor environment: *F*_1,12_ = 24.0, *p* < 0.001). As was mentioned previously, a negative correlation between epistasis and mutation effect size can result from measurement error alone [10], but correcting our data for effects from measurement error resulted in slightly stronger negative correlations (see the electronic supplementary material).

To remedy our limited ability to detect sign epistasis, we used a maximum-likelihood approach to fit FGM to the two datasets, which takes into account the contribution of sign epistasis through a survival analysis for left-truncated data (see the electronic supplementary material and [31]). In brief, we derived the analytical results for the mean and variance of epistasis conditioned on the two single effect sizes being equal*,* and used them to construct the distribution of epistasis under a Gaussian approximation for the intrinsic variability of FGM*.* To incorporate the instances where sign epistasis is present but not observed, the distribution is modified through a survival analysis, which implies that the integrated probability density of the truncated region (*ɛ* < −*s*) is assigned as a statistical weight to all points located below the line *ɛ* = −*s*. Standard measurement error is modelled as an additional extrinsic Gaussian random noise with a variance determined from the replicate measurements. Furthermore, cases where sign epistasis can be detected are accounted for through a separate parameter *q* that quantifies the probability that an observation below the line *ɛ* = −*s s*hould be attributed to fast germination among sign epistatic pairs (when the double mutant germinates fast and in this way prevents the linear outgrowth of the single mutants), rather than to measurement error. Finally, the principle of maximum-likelihood is applied to estimate the parameters of the complete statistical model, which thus includes experimental uncertainties as well as the intrinsic variability of FGM on an equal footing.

As shown in figure 3*b*, FGM describes the diminishing-returns pattern in both environments rather well. Importantly, although the fit of *mean* epistasis by FGM is not significantly better than a simple linear relationship, it explains a much larger proportion of the data, because it accounts for intrinsic variation in epistasis, whereas a linear model does not. This claim is supported by the fact that 98% and 94% of the data points, respectively for the rich and poor environment, lie in the 99% variability region derived from FGM, whereas only 57% and 54% lie within the variability region defined by measurement error around the best-fitting linear model (see the electronic supplementary material). The model fit predicts similar fitness distances to the optimum for the rich and poor environment (*s*_0_/*s*_m_ = 1.41 versus 1.62, respectively, where *s*_m_ is the largest single mutant selection coefficient observed in the respective datasets), but a lower mutational distance to the optimum (*ρ* = 6.89 versus 9.81, respectively) and lower phenotypic dimensionality (*n* = 19.3 versus 34.8, respectively) for the rich relative to the poor environment. It may seem surprising that we find a lower phenotypic dimension in the rich medium with its greater diversity of carbon sources. However, theoretical studies of FGM show that the correlation between the phenotypic dimension and the ruggedness of the induced genotypic fitness landscape is often quite weak, and certain ruggedness measures in fact decrease with increasing *n* [27] (Hwang *et al*. 2016, unpublished results). The probability of fast germination among sign epistatic pairs (*q*) inferred from the data is low in the rich environment (*q* = 0.21) and high in the poor environment (*q* = 1). This reflects the fact that most of the data points in the sign epistatic region *ɛ* < −*s* are close to the line *ɛ* = −*s* for the rich medium, and thus should be attributed to measurement error, whereas only two such instances are found in the poor medium data, one of which is far below the line and hence likely owing to fast spore germination.

We performed two further tests of the ability of the parametrized version of FGM to describe the observed pattern of epistasis. First, we used FGM to predict the frequency of sign epistasis among beneficial mutations in the rich and poor environment. Here, the empirical frequency of sign epistasis is estimated by summing the corresponding probabilities derived from the parameterized FGM over all experimental data points. Figure 3*c* shows the predicted probability distributions for the frequency of sign epistasis in both environments (derived in the electronic supplementary material), indicating 16 of the 41 pairs expected in the rich environment and two of the 13 pairs expected in the poor environment. The empirical estimates coincide with the predicted expectation values in both cases. Second, from the versions of FGM parametrized with the epistasis estimates, we inferred the distribution of fitness effects for the sets of beneficial mutations that were initially isolated and used in the crosses (see the electronic supplementary material). As shown in figure 2*b*, this model describes the observed distributions remarkably well, and not worse than when the model parameters are estimated directly from these data using a maximum-likelihood analysis based on the single effect size distribution predicted by FGM (see electronic supplementary material and [24]). In fact, the inference using the epistasis data is more stable, because the log-likelihood function derived from the single effect size distribution is very flat in the relevant region of parameter space (see [32] for a discussion of related issues).

## 3. Perspective

Our results confirm recent reports of negative epistasis among beneficial mutations and its dependence on the selection coefficient of mutations in single genes and unicellular microbes [2,11,12,14]. Based on analyses of epistasis between independently selected genome-wide beneficial mutations, we show that this pattern is generic and holds also for a multicellular organism. Previous studies have shown the ability of FGM to predict the distribution of epistasis among pairs of mutations [16,26,33] and the negative correlation between epistasis and fitness of the genetic background [13,27]. Vice versa, genotype-fitness landscapes inferred from a nonlinear phenotype-fitness map similar to FGM have been shown to accurately describe empirical fitness landscapes for independently selected beneficial mutations [14]. Simple statistical genotype-fitness models, such as the house-of-cards model [34], also predict diminishing-returns epistasis for independent beneficial mutations. However, such models describe the data much more poorly (see electronic supplementary material, figure S5), and are not informative about possible biological causes of epistasis.

By demonstrating how a specific relationship between mutation-effect size and epistasis can emerge from a nonlinear phenotype-fitness map, our analyses exemplify the use of FGM as a proof-of-concept model [35]. The predictive power of FGM is remarkable in the light of the simplifying and even incorrect assumptions it makes. For example, in contrast to assumptions of FGM, mutations often have non-additive phenotypic effects [36], multiple phenotypic optima may exist [37], and not all phenotypes necessarily affect fitness equally [38]. Its utility, however, does not only depend on its accuracy to predict patterns of epistasis, but also on how much it can inform about the mechanisms causing epistasis. Whereas phenotype-fitness models based on biochemical [36] or biological processes are directly informative [1], it has been argued also that FGM emerges from general first principles describing metabolic networks and developmental processes [22]. Moreover, FGM provides a natural mechanism for generating intrinsic stochasticity in the effects of single and multiple mutations, which turned out to be crucial for explaining the variability in our data. The growing support for diminishing-returns epistasis among beneficial mutations, which is predicted by FGM, may thus not only explain the declining rates of adaptation repeatedly seen in evolution experiments [2], but also point at the underlying causes.

## 4. Experimental methods

### (a) Experimental system and construction of starting genotypes

We used the filamentous fungus *A. nidulans* as a model system. *A. nidulans* is a non-pathogenic soil-borne fungus that is widely used for genetic and evolutionary studies [28,30,39–42]. For the construction of strains for this study, we started with strains WG652 (*fld*A1, *lys*B5) and WG653 (*fld*A1, *ribo*B2) from the Wageningen strain collection where all strains have originated from the same genetic background. Strains that carry the *fld*A1 marker have reduced fitness when growing on regular complete medium (CM) [43]. Previous work has shown that a multitude of adaptive routes and mutational targets exist for strains of this genotype to adapt to the imposed suboptimal conditions [44,45].

We crossed strains WG652 and WG653 and selected progeny with genotype *fld*A1, *ribo*B2, which we backcrossed to WG652. After seven repeated backcrosses, we selected two progeny, one with genotype *fld*A1, *lys*B5 and one with genotype *fld*A1, *ribo*B2 (called strain A0 and strain B0). Once the growing medium is supplemented with the compounds the strains are deficient for (in this case, lysine and riboflavin), there is no detectable fitness effect of the selectable markers. In this way, we generated two strains that are more than 99.99% genetically identical and differ in one selectable marker. Selectable markers facilitate sexual crosses between the strains: two strains can be forced to grow as a mycelium composed of two types of nuclei (i.e. as a heterokaryon) when grown on minimal medium (MM) not supplemented with the essential growth factors the strains are deficient for (lysine and riboflavin). In this way, the likelihood is greatly increased that two non-identical nuclei fuse to form a zygote that will lead to outcrossing [29]. We set up a cross with strains A0 and B0 and assayed fitness of 40 random progeny across a range of five environments, and did not detect fitness variation among the progeny (GLM, *F*_12,59_ = 1.34, *p* = 0.22).

### (b) Growth media, conditions and fitness measurements

We used Petri dishes with either 25 ml of solid rich medium (CM) or poor medium (MM). Both media are set at pH = 5.8 and consist of NaNO_3_ 6.0 g l^−1^, KH_2_PO_4_ 1.5 g l^−1^, MgSO_4_ · 7H_2_O 0.5 g l^−1^, NaCl 0.5 g l^−1^, 1.0 mg of each of the trace elements FeSO_4_, ZnSO_4_, MnCl_2_ and CuSO_4_ and (added after autoclaving) sucrose 4.0 g l^−1^; riboflavin 0.1 mM and lysine 0.1 mM. In addition to this, CM also contains tryptone 10 g l^−1^ and yeast extract 5 g l^−1^. Cultures were incubated at 37°C. For sexual crosses, we used crossing minimal medium (cMM), consisting of NaNO_3_ 1.0 g l^−1^, KH_2_PO_4_ 1.5 g l^−1^, MgSO_4_ · 7H_2_O 0.5 g l^−1^, KCl 0.5 g l^−1^, 1.0 mg of each of the trace elements FeSO_4_, ZnSO_4_, MnCl_2_ and CuSO_4_, glucose 20 g l^−1^, agar 15 g l^−1^, with fludioxonil 20 ppm, and pH set at 5.8.

Fitness was measured by placing 5 µl of a dense spore suspension (more than 10 000 spores) in the centre of a Petri dish containing either rich or poor medium. After 5 days of growth, the colony diameter was measured in two perpendicular directions. Linear growth has been shown to be a good proxy for fitness, based on its positive correlation with other fitness measures such as spore production rate and competitive fitness [44,46–49], however it does not correlate with spore germination speed.

### (c) Generation of mutants carrying one beneficial mutation

We inoculated a total of 1438 Petri dishes for strain A0 and for strain B0 by placing 5 µl of a dense spore suspension in the centre of a Petri dish containing solid rich CM or poor MM (614 plates of CM and 824 plates of MM). After 5 days of growth, we collected mycelium and spores at the growing edge of the colony. We used this material to measure fitness in triplicate by quantifying the mycelium growth rate (MGR) as the size of a colony founded from the centre of a Petri dish after 5 days of (linear) growth and compared this with the fitness of the starting genotype. Mycelia with fitness significantly higher than the ancestor (i.e. higher than the 99.99% upper limit of the confidence interval) were considered to carry a beneficial mutation. We identified 154 such strains collected from rich CM medium and 90 from poor MM. Note that we also picked up mutants with a lower fitness than the ancestor. This could be attributed to a trade-off between germination speed and mycelial growth rate, to drift effects where a recessive deleterious mutation hitchhikes along in the multinuclear mycelium, or to measurement error.

Recently, whole genome sequence data became available for eight experimental evolution lineages founded by the same strain as used in this study (Dettman *et al.* 2016, unpublished data). These experimental evolution lines had undergone eight rounds of selection [44], each round being similar to our procedure of generating single beneficial mutations. The genome sequences show that on average 10.38 (s.e. = 1.27) mutations (including SNPs, indels and re-arrangements) accumulated, of which 6.88 (s.e. = 1.04) were non-synonymous mutations in coding regions. This implies that in our experimental set-up we expect lineages to have on average 6.88/8 = 0.86 mutations (s.e. = 0.13) that may affect fitness, consistent with expectations that each strain contains a single mutation. Moreover, the WGS data show that no mutations were found in the same gene in more than one strain, supporting their genome-wide occurrence and limited likelihood that the same mutation occurred in both parents in our crosses. Although we cannot exclude the possibility that more than one mutation was present in any of the parental strains, the conclusion that beneficial mutations show diminishing-returns epistasis remains unaffected. In such case, the epistasis observed involves that between sets of two mutations, or perhaps between single and double mutations. This would affect the scale of ‘mutations’ for which we consider epistasis, but because the relationship predicted by FGM is monotonic (figure 3), it would at most affect the absolute value of epistasis, not the fact that it is negative on average and shows a diminishing-returns relationship (see the electronic supplementary material).

### (d) Construction of double mutants

We assigned the pairs of single mutants with similar fitness effects, using one mutant from the A background and one from the B background. To do so, we ranked all mutants from the A and the B derived strains based on their fitness. Strains with the same rank number were used to construct the double mutants. We calculated the average difference in MGR between the two single mutants used to form a double mutant. This was on average 1.11 mm (s.d. = 1.86). The fitness of the ancestors is 35 mm, so the pairs differed on average 3.1% at the level of ancestral fitness. We used each beneficial mutant in one unique combination to avoid pseudo-replication. We set up sexual crosses, following standard protocols [29]. Briefly, we grew heterokaryons of the two strains on cMM. Within this heterokaryon, the *A. nidulans* sexual cycle starts when two haploid nuclei form a zygote. This zygote multiplies through a series of mitoses, after which each zygote goes through meiosis. This results in sexual fruiting bodies containing up to 10^6^ sexual spores (ascospores) representing all possible recombinants of meiosis [29,50,51]. Among the progeny, we expect two parental and two recombinant classes (i.e. the double mutant and the ancestral type with no mutation), assuming no genetic linkage between mutations, and these fitness classes should appear in an equal ratio (1 : 1 : 1 : 1). Sexual fruiting bodies from heterokaryons were collected and crushed in 100 µl saline–Tween (0.8% NaCl and 0.005% Tween-80 in water) and checked for being products of outcrossing between the two strains by plating on MM without lysine and riboflavin. We spread 70 µl of the undiluted progeny mix on MM to eliminate the auxotrophic makers, to avoid measuring any potential epistatic fitness effects of the markers on the beneficial mutations. Further, we obtained a dense spore suspension representative of all progeny by washing down the Petri dish after 3 days of growth for further analysis and for long-term storage at −80°C (after adding 300 µl 80% glycerol solution to 700 µl of the spore suspension).

### (e) Fitness measurements of double mutants to estimate epistasis

Fitness of the double mutant was estimated using the fitness of the collective of spores in sexual fruiting bodies alongside with measuring fitness of the three known genotypes in each combination (the two single mutants and the ancestor without mutations) for 55 crosses (42 CM + 13 MM). Based on previous work, we expect the progeny genotype in the mixture with the highest fitness to determine the outcome of the fitness assay, because its growth rate will dominate the growth rate of the collective. If the double mutant has the highest fitness, we are able to accurately measure fitness of all four classes. If one of the single mutants has the highest fitness (so, in case of sign-epistasis), we will not be able to determine the fitness of the double mutant.

In some cases, we observed that the mixture of all progeny had a lower fitness than at least one of the single-mutation parents. Because this was unexpected, we repeated the assay with dilutions of the mix of progeny, and found that when diluting to 10 spores or less per inoculum, this effect disappears. We reasoned that these double mutants must have a faster spore germination rate than the other three genotypes, combined with a slower growth rate. The fast germination gives the double mutants an initial advantage of occupying space and thereby preventing the other genotypes to leave the inoculation area, consistent with observations by Gifford *et al*. for this fungus [30].

Epistasis (*ɛ*) was quantified as the deviation of observed fitness of the double mutant (Δ*f_ab_*) from the expectation of additive fitness effects of the single mutants (Δ*f_a_* and Δ*f_b_*). Hence, epistasis is calculated in terms of the actual growth rate, so our epistasis measure has the units millimetres per 5 days. Because fitness is determined by the linear growth rate, we use an additive rather than a multiplicative model to calculate *ɛ*: *ɛ* = Δ*f_ab_* − (Δ*f_a_* + Δ*f_b_*) [16,18,28].

We chose not to isolate single progeny from each cross and to estimate epistasis from single progeny measurements. This method would have the disadvantage that several purification steps are needed during which additional mutations could arise. The advantage of the ‘collective method’ we used is that a limited number of cultivation steps are needed and that the assays remain small making them more accurate and allow measurements in one block. The limitation of our method of not being able to accurately detect sign-epistasis is dealt with by considering expected frequencies of sign epistasis from FGM during model fitting (see section IC1 of the electronic supplementary material).

## Supplementary Material

Mathematical Supplement

## Data accessibility

Data have been deposited in dryad doi:10.5061/dryad.6fb43. Scripts are available upon request.
