# Supplementary material for: Diminishing-returns epistasis among random beneficial mutations in a multicellular fungus
Source: Proc Biol Sci. 2016 Aug 31;283(1837):20161376. doi: 10.1098/rspb.2016.1376 (PMC5013798; doi:10.1098/rspb.2016.1376)
Supplement: Mathematical Supplement [file rspb20161376supp1.pdf]

Mathematical supplement

CONTENTS

I. Fisher's geometric model 1
A. Model description 1
B. Epistasis 2
1. Definition 2
2. Moments of epistasis and Gaussian approximation 3
3. Sign epistasis 4
4. Multiple mutations in one strain 5
C. Data analysis 6
1. Inferring model parameters 6
2. Estimating the frequency of sign epistasis 7
3. Comparison with a linear model 8
4. Parameter estimation from single effect distribution 8
5. A correction to the Pearson correlation coefficient due to measurement error 9

II. House of cards model 10

References 10

I. FISHER'S GEOMETRIC MODEL

A. Model description

Fisher's geometric model (FGM) is a phenotypic fitness landscape model where the organism is described by an n-dimensional phenotype $\vec{x} = (x_1, x_2, \cdots, x_n)$ in the real Euclidean vector space $\mathbb{R}^n$ and fitness is defined as a smooth, single-peaked function $f(\vec{x})$ of $\vec{x}$ [1, 2]. The dimension n is often referred to as phenotypic complexity and can be interpreted as the number of 'optimized' traits that contribute to fitness [3]. By translational invariance, the optimal phenotype may be placed at the origin.

As the function $f(\vec{x})$ is assumed to be smooth, there exists a region around the optimum where it can be well approximated by a parabola. Since a living organism is believed to be well-adapted, we assume that the wild-type phenotype lies inside this region. Furthermore, if each trait determines the fitness equally strongly and independently, we may additionally assume that f is isotropic. In fact, it was recently shown in [3] that the isotropic FGM emerges from first principles based on random matrix theory under fairly general conditions. Summing up all the assumptions, the fitness is given by

$$f(\vec{x}) = f_0 - \Lambda|\vec{x}|^2 = -\Lambda|\vec{x}|^2, \tag{1}$$

where $|.|$ is the Euclidean norm and Λ determines the curvature of the fitness function around the origin. Here, f_0 is set to zero since an overall additive constant does not affect any statistical properties. Note that, in contrast to many previous studies of FGM [2], we adopt a Malthusian rather than a Wrightian fitness concept. Consistency with previous results can however always be achieved by interpreting f as the logarithm of a Wrightian fitness function W.

The fixation of a mutation i in the population corresponds to a jump from the wildtype phenotype $\vec{x}_0$ to the mutant phenotype $\vec{x}_i$ and is thus described by a displacement vector $\vec{\Delta x}_i \equiv \vec{x}_i - \vec{x}_0$. A basic assumption in the applications of FGM to the study of epistasis [2, 4, 5] is the absence of epistatic interactions on the level of the phenotype, which implies that the induced phenotypic change due to a given mutation i is independent of the wildtype phenotype $\vec{x}_0$. Thus the statistical properties of mutations are described by the distribution of displacement vectors, which is usually assumed to be an n-dimensional Gaussian distribution,

$$M(\vec{\Delta x}) = \frac{e^{-|\vec{\Delta x}|^2/2\sigma^2}}{\sqrt{(2\pi\sigma^2)^n}}, \tag{2}$$

where the standard deviation σ quantifies the typical size of the phenotypic displacements. The specific form (2) can be justified using central limit arguments if the number of optimized traits is much smaller than the total number of phenotypic traits affected by mutations [3].

From the description of the model, we have two basic phenotypic scales: the distance $r_0 = |\vec{x}_0|$ of the wildtype from the optimum and the typical size σ of mutational displacements. Their ratio will be denoted by $\rho = r_0/\sigma$ (see Fig. 1 A of the main text). Rescaling the phenotype by r_0 we introduce $\vec{\lambda} = \vec{x}/r_0$ as dimensionless coordinates. Moreover, since the fitness function f is isotropic, the wildtype position can be taken to lie on the first coordinate axis, i.e., $\vec{x}_0 = r_0\hat{e}_1$ and $\vec{\lambda}_0 = \hat{e}_1 \equiv (1, 0, 0, \cdots, 0)$. In the new variables, the distribution of phenotypes of a single mutant is given by

$$Q(\vec{\lambda}) = \frac{\rho^n}{(2\pi)^{n/2}} \exp\left(-\frac{\rho^2}{2}[(\lambda_1 - 1)^2 + \lambda_2^2 + \cdots + \lambda_n^2]\right) \tag{3}$$

or, in spherical coordinates $(\lambda = |\vec{\lambda}|, \theta)$,

$$Q(\lambda, \theta) = \frac{S_{n-2}}{(2\pi)^{n/2}} \rho(\rho\lambda)^{n-1}(\sin\theta)^{n-2} \exp\left(\frac{-\rho^2}{2}(\lambda^2 - 2\lambda\cos\theta + 1)\right), \tag{4}$$

where S_n is the surface area of the unit n-sphere. Integrating further over θ, the marginal distribution of the radial distance is given by

$$\bar{Q}(\lambda) = \lambda^{n/2}\rho^2 \exp\left(-\frac{\rho^2(1+\lambda^2)}{2}\right) I_{\frac{n-2}{2}}\left(\rho^2\lambda\right), \tag{5}$$

where $I_k(x)$ is a modified Bessel function of the first kind.

Similarly, the fitness function (1) is rescaled as above to give

$$f(\lambda) = -\lambda^2 s_0 \tag{6}$$

where we introduce another parameter $s_0 = \Lambda r_0^2$ describing the selection strength of the optimum or the largest single mutational effect that can be achieved from the wild type (Fig. 1A). The (Malthusian) selection coefficient s is then related to λ as

$$s \equiv f(\lambda) - f(\lambda = 1) = (1 - \lambda^2)s_0. \tag{7}$$

Using (7), the distribution (5) can be written in terms of s, yielding the expression

$$Q_{\rm sel}(s) = \frac{1}{2}\rho^2 s_0^{-\frac{n}{4}-\frac{1}{2}} (s_0 - s)^{\frac{n-2}{4}} e^{\frac{\rho^2(s-2s_0)}{2s_0}} I_{\frac{n-2}{2}}\left(\rho^2\sqrt{\frac{s_0 - s}{s_0}}\right), \tag{8}$$

which was also reported in [2].

B. Epistasis

1. *Definition*

In the following, we investigate the statistical properties of the pair-wise epistasis ϵ, a measure quantifying the fitness correlation between a pair of mutations. To define this quantity, suppose we have two mutants corresponding to phenotypic displacements $\vec{\Delta x}_1 = r_0\vec{\Delta\lambda}_1$ and $\vec{\Delta x}_2 = r_0\vec{\Delta\lambda}_2$. Then the dimensionless phenotypes of the mutants are given by $\vec{\lambda}_i = \vec{\lambda}_0 + \vec{\Delta\lambda}_i$, where $\vec{\lambda}_0 = \hat{e}_1 \equiv (1, 0, 0, \cdots, 0)$ in the rescaled space. Because there are no epistatic interactions on the level of phenotypes, the position of the double mutant is similarly written as $\vec{\lambda}_{12} = \vec{\lambda}_0 + \vec{\Delta\lambda}_1 + \vec{\Delta\lambda}_2 = \vec{\lambda}_1 + \vec{\lambda}_2 - \vec{\lambda}_0$ (see Fig. 1B). As a measure for mutational interaction defined in [4], the pairwise epistasis quantifies the deviation of the double mutational effect from the additive null model, i.e.,

$$\begin{aligned}\epsilon &\equiv \left[f(|\vec{\lambda}_{12}|) - f(|\vec{\lambda}_0|)\right] - \left[f(|\vec{\lambda}_1|) - f(|\vec{\lambda}_0|)\right] - \left[f(|\vec{\lambda}_2|) - f(|\vec{\lambda}_0|)\right] \\ &= f(|\vec{\lambda}_{12}|) - f(|\vec{\lambda}_1|) - f(|\vec{\lambda}_2|) + f(|\vec{\lambda}_0|) \\ &= -2s_0(\vec{\lambda}_1 - \hat{e}_1)\cdot(\vec{\lambda}_2 - \hat{e}_1) = -2s_0\Delta\vec{\lambda}_1\cdot\Delta\vec{\lambda}_2.\end{aligned} \tag{9}$$

Since the phenotype displacement is a random variable in this model, the pairwise epistasis (9) is random as well. In our experimental setting, we are mostly interested in the situation where two mutants share the same single effect s or equivalently λ. Under this condition, we want to understand the statistical properties of ϵ. Obviously, to fully characterize the behavior of ϵ, its distribution $P_\lambda(\epsilon)$ conditioned on λ would be needed. However, finding the full distribution seems to be quite challenging mathematically. Instead, we shall focus on obtaining the first two cumulants, i.e., the mean and the variance of $P_\lambda(\epsilon)$.

2. *Moments of epistasis and Gaussian approximation*

As mentioned above, the epistasis defined in (9) inherits its randomness from the mutational displacements which are distributed according to the distribution $Q(\lambda, \theta)$. To distinguish the two averages involved, it is convenient to introduce distinct notations for them. For the averages with respect to the displacement distribution $Q(\lambda, \theta)$ conditioned on fixed λ, we use angular brackets such that

$$\langle \cdots \rangle_\lambda \equiv \frac{1}{Z_\lambda} \int d\theta \, (\cdots) Q(\lambda, \theta) \tag{10}$$

where Z_λ is the normalization constant defined by

$$Z_\lambda \equiv \int d\theta \, Q(\lambda, \theta). \tag{11}$$

Similarly, $\mathbb{E}_\lambda$ is used for the expectation with respect to $P_\lambda(\epsilon)$. Using these notations, the following expressions can be derived for the first two moments of ϵ,

$$\mathbb{E}_\lambda(-\epsilon/2s_0) = \langle \lambda \cos\theta - 1 \rangle_\lambda^2 \tag{12}$$

and

$$\mathbb{E}_\lambda(\epsilon^2/4s_0^2) = \left\langle (\lambda \cos\theta - 1)^2 \right\rangle_\lambda^2 + \frac{1}{n-1} \left\langle (\lambda \sin\theta)^2 \right\rangle_\lambda^2, \tag{13}$$

for $n \geq 2$. As an immediate consequence of (12) it is seen that the mean epistasis is always negative. A similar result was obtained in [5] for beneficial mutations of unconstrained effect size.

After evaluating the expressions (12) and (13) by integrating the distribution (4) over θ one finds

$$\mathbb{E}_\lambda(-\epsilon/2s_0) = \left(\frac{\lambda I_{\frac{n}{2}}\left(\lambda\rho^2\right)}{I_{\frac{n-2}{2}}\left(\lambda\rho^2\right)} - 1 \right)^2 \tag{14}$$

and

$$\begin{aligned}\mathbb{E}_\lambda(\epsilon^2/4s_0^2) = & \frac{\lambda^2 (n-1) I_{\frac{n}{2}}\left(\lambda\rho^2\right)^2}{\rho^4 I_{\frac{n-2}{2}}\left(\lambda\rho^2\right)^2} \\ & + \left(\frac{\lambda^2 \left(\frac{I_{\frac{n}{2}}\left(\lambda\rho^2\right)}{\lambda\rho^2} + I_{\frac{n+2}{2}}\left(\lambda\rho^2\right) \right)}{I_{\frac{n-2}{2}}\left(\lambda\rho^2\right)} - \frac{2\lambda I_{\frac{n}{2}}\left(\lambda\rho^2\right)}{I_{\frac{n-2}{2}}\left(\lambda\rho^2\right)} + 1 \right)^2,\end{aligned} \tag{15}$$

for $n \geq 1$ [6]. The limiting behaviors of $\mathbb{E}_\lambda(-\epsilon/2s_0)$ in the two limits $\rho \to 0$ and $\rho \to \infty$ are

$$\mathbb{E}_\lambda(-\epsilon/2s_0) = \begin{cases} 1 + O(\rho) & \text{for} \quad \rho \to 0 \\ \left(1 - \lambda + \frac{n}{2\rho^2} + O(\rho^{-2})\right)^2 & \text{for} \quad \rho \to \infty. \end{cases} \tag{16}$$

This suggests that n and ρ enter the expression for the mean epistasis only in the combination $z = \sqrt{n}/\rho$. Even though all the results have been derived in terms of λ for the sake of simplicity, one can always replace λ by the selection coefficient s via the relation (7), which will be more useful when analyzing experimental data. The predicted

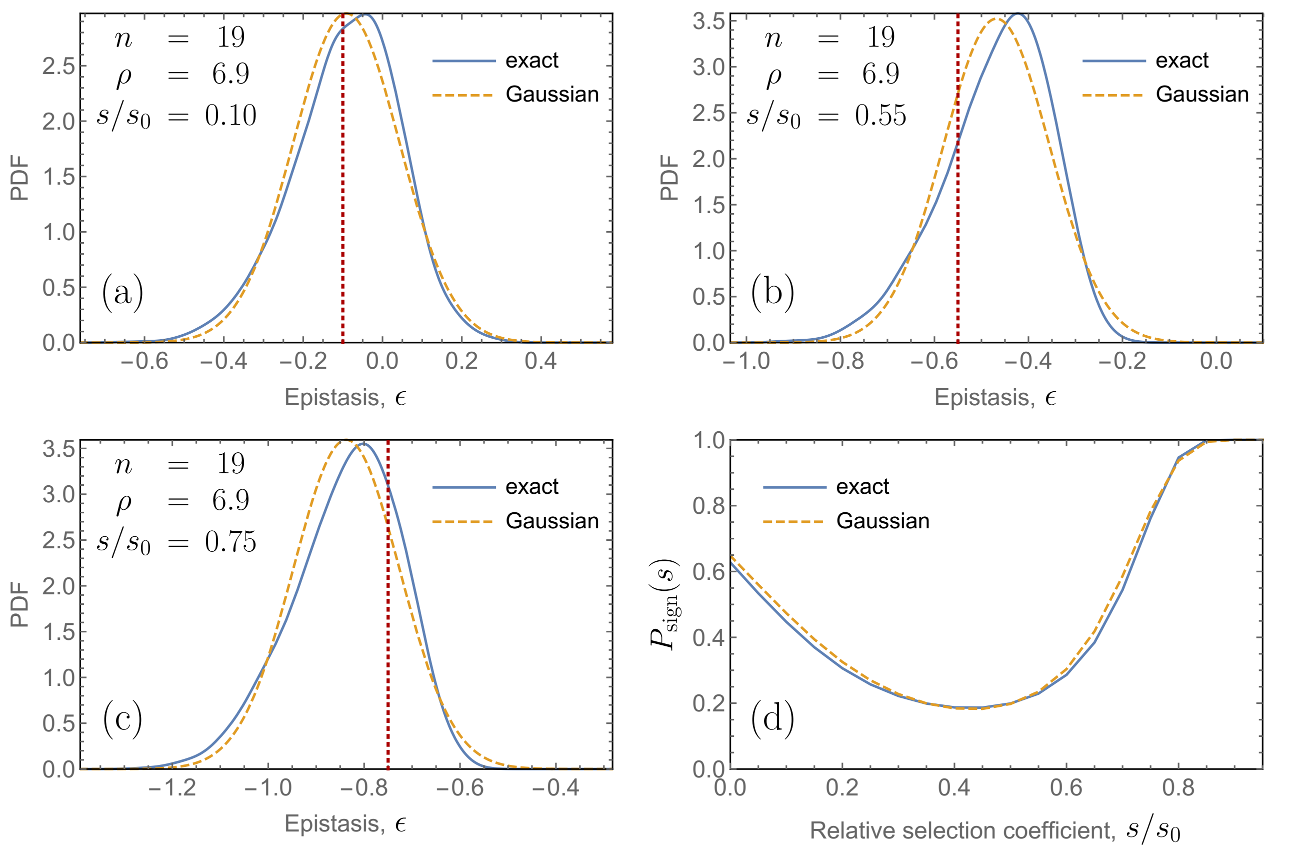

FIG. S1. A comparison between the exact distribution $P_\lambda(\epsilon)$ of epistasis, obtained using simulations, and its Gaussian approximation for different selection coefficients. The other two parameters n and ρ are chosen such that they are close to the parameters estimated from the data. The vertical dotted line indicates the onset of sign epistasis which is given by $-s$. Then, the probability of sign epistasis is determined by the integral of $P_\lambda(\epsilon)$ over the interval $(-\infty, -s)$ [panel (d)].

dependence of mean epistasis on the selection coefficient is illustrated in Fig. 1C of the main text. Numerical results for this dependence were also reported in [5].

Although the full distribution $P_\lambda(\epsilon)$ of epistasis cannot be obtained in a closed form, it can be approximated by a Gaussian distribution using the expressions (14) and (15) for the first two cumulants. As shown in Fig. S1, this approximation works well in the parameter range of interest here. However, readers should be warned that it is no longer valid for small n as can be easily checked from the $n = 1$ case where the distribution becomes discrete with three possible values.

3. Sign epistasis

As was explained in the main text, sign epistasis, where a given mutation can be beneficial or deleterious depending on the genetic background [7], takes a particularly simple form in the present setting. When both single mutants have the same positive selection coefficient s, sign epistasis occurs whenever the fitness of the double mutant is lower than that of the single mutants, which according to the definition (9) amounts to

$$\epsilon < -s. \tag{17}$$

Because the fitness effects of the single mutants are identical, sign epistasis is always reciprocal in this case [8].

The propensity for sign epistasis can be qualitatively inferred from the shape of the mean epistasis curve in Fig. 1C of the main text. Consider first the situation where the wildtype phenotype is far from the optimum (large ρ). Then the mean epistasis curve lies mostly above the line $\epsilon = -s$ and sign epistasis is rare. As ρ decreases the curve straightens and becomes more negative, indicating the increased ‘ruggedness’ of the genotype landscape closer to the phenotypic optimum. Finally, for $\rho \to 0$, $\mathbb{E}_\lambda(\epsilon) \to -2s_0$ which is below the line $\epsilon = -s$ for all s (recall that $s \leq s_0$ by construction).

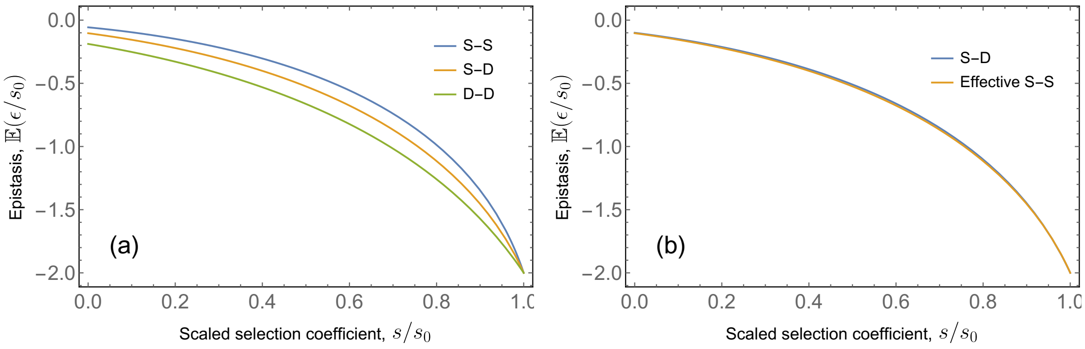

FIG. S2. (a) Plot of mean epistasis between two mutants carrying different numbers of mutations; S stands for single mutant, D for double mutant, and the FGM parameters are $n = 19$ and $\rho = 7$. The introduction of multiple mutations effectively changes the length scale ρ (compare to Fig.1C in the main text where the variation of mean epistasis with ρ is displayed). (b) Comparison of mean epistasis in the S-D case with the S-S expression evaluated for an effective parameter $\rho_{\text{eff}} = \frac{1}{2}(\rho + \rho/\sqrt{2})$.

Interestingly, the concave shape of the function (14) suggests that, as ρ decreases, the regions where $\mathbb{E}_\lambda(\epsilon) < -s$ appear at small and large s. This tendency is further amplified by the behavior of the variance of ϵ. Over the range of s ($0 < s < s_0$), one can show that the variance (15) is smallest at an intermediate point s_i ($0 < s_i < s_0$) and increases as s deviates from that point in the parameter ranges of our interest. The nonmonotonic variation of the probability for sign epistasis as a function of s is indeed confirmed by the direct evaluation of this quantity in Fig. S1(d). It accounts for two distinct mechanisms by which (reciprocal) sign epistasis may arise in FGM: For mutations of large effect it occurs by overshooting the optimum, while for mutations of small effect the underlying mechanism is antagonistic pleiotropy [5].

4. Multiple mutations in one strain

In the analysis of the data reported in the main text it is assumed that all strains that were crossed to generate double mutants each carry a single mutation on the background of the wild type. As explained in the *Experimental Methods* section, however, the probability that strains may harbor more than one mutation was estimated to be small but not entirely negligible. In the following we show that the intepretation of the data within the framework of FGM is only weakly affected by the occurrence of multiple mutations in one strain.

In FGM, mutations are described by Gaussian random displacements. Hence, when two mutations occur, we can effectively replace the combined effect by a single displacement but with a size differing by a factor of $\sqrt{2}$ (recall that the sum of two Gaussian random variables is again a Gaussian random variable with twice the variance). Following a calculation similar to the one performed to obtain (14), we can derive the mean epistasis between mutations that consist of different numbers of elementary mutational displacements. Specifically,

$$\mathbb{E}_\lambda^{D-D}(-\epsilon/2s_0) = \left(\frac{\lambda I_{\frac{n}{2}}\left(\frac{\lambda\rho^2}{2}\right)}{I_{\frac{n-2}{2}}\left(\frac{\lambda\rho^2}{2}\right)} - 1 \right)^2 \tag{18}$$

for the case where both mutant strains carry two mutations each and

$$\mathbb{E}_\lambda^{S-D}(-\epsilon/2s_0) = \left(\frac{\lambda I_{\frac{n}{2}}\left(\lambda\rho^2\right)}{I_{\frac{n-2}{2}}\left(\lambda\rho^2\right)} - 1 \right) \times \left(\frac{\lambda I_{\frac{n}{2}}\left(\frac{\lambda\rho^2}{2}\right)}{I_{\frac{n-2}{2}}\left(\frac{\lambda\rho^2}{2}\right)} - 1 \right) \tag{19}$$

for the case where one strain carries two mutations while the other carries one. As shown in Fig. S2, this variation introduces a slight rescaling of ρ but does not change the overall behavior significantly. This implies that, in the presence of multiple mutations, the estimate of ρ from the data should be considered as an effective parameter value that is slightly smaller than the true one [see Fig. S2(b)].

C. Data analysis

1. Inferring model parameters

In order to test our analytical predictions (14) and (15), the following analysis was performed on the data collected from the experiment with *Aspergillus nidulans* described in the main text. First, among the beneficial mutations, we selected pairs of single mutants having similar effects ($f_A \simeq f_B$). Exploiting the sexual cycle of the fungus for each pair of mutations, a fruiting mass was formed that contains all four possible genotypes (W, A, B, AB). Subsequently, we performed a growth assay in which the most fit among the four genotypes is expected to dominate the population.

As we initially picked two beneficial mutations satisfying $f_A \simeq f_B > f_W$, the dominating population will be either the double mutant AB or one of the single mutants. In other words, the genotype AB will dominate the population unless sign epistasis occurs, i.e, provided that $\max\{f_A, f_B\} < f_{AB}$. Otherwise, there are two possible scenarios that can emerge: i) one of the single mutants will dominate and the fitness of the double mutant (and hence the value of ϵ) is not observable or ii) the double mutant will dominate despite being of lower fitness because of a tradeoff between fast germination and slow growth, see the *Methods* section in the main text.

To account for this experimental situation, it is convenient to define the pseudo-epistasis

$$\epsilon' = \max\{f_{AB}, f_A, f_B\} - f_A - f_B + f_W. \tag{20}$$

It can be checked that $\epsilon' = -f_A + f_W = -s$ for the case that one of the single mutants dominates while it is equal to the true epistasis ϵ otherwise, that is,

$$\epsilon' = \max\{\epsilon, -s\}. \tag{21}$$

Additionally, we introduce the probability q that the double mutant is observed when the true sign epistasis occurs , which is possible only if the double mutant germinates fast and prevents growth of the single mutants. In this case the measured epistasis is the true epistasis (plus the measurement error) instead of the pseudo epistasis. The measurement errors are modeled by a Gaussian distribution with mean zero and variance σ^2_{Error}. The variance σ^2_{Error} is determined to be the average of the unbiased estimators of variance, each calculated from up to six replicates of the corresponding data point. Hence, its probability density function $P_e(\xi)$ is given by

$$P_e(\xi) = \frac{e^{-\frac{\xi^2}{2\sigma^2_{\text{Error}}}}}{\sqrt{2\pi}\sigma_{\text{Error}}} \tag{22}$$

and each measured epistasis value m is modeled as the sum $\xi + \epsilon$ with probability q and $\xi - s$ with probability $(1-q)$. As a consequence of the measurement error, the data points corresponding to pairs which display sign epistasis are seen to be mildly scattered around the line $\epsilon = -s$ in Fig. 3B of the main text, whereas those cases where the data points are located significantly below this line are attributed to fast germination.

The inference of the FGM parameters n, ρ and s_0 from the pairwise epistasis data requires a suitable choice of a log-likelihood function $\mathcal{L}$. To construct this function, one should first calculate the probability density function (PDF) $P_{\text{tot}}(m|s, \mathcal{P})$ of the measured epistasis m conditioned on s and on the model parameters $\mathcal{P}$. Here, $\mathcal{P} = \{n, \rho, s_0, q\}$ is the set of the model parameters of FGM. Since the measured epistasis is modeled as the sum of two independent random variables, one arising from the intrinsic stochasticity of FGM and the other from measurement error, the resulting PDF $P_{\text{tot}}(m)$ is written as the convolution of both variables, i.e.,

$$P_{\text{tot}}(m|s, \mathcal{P}) = (P_{\text{FGM}} * P_e)(m|s, \mathcal{P}) \equiv \int_{-\infty}^{\infty} P_{\text{FGM}}(m - \xi|s, \mathcal{P}) P_e(\xi)\, d\xi, \tag{23}$$

where $P_{\text{FGM}}(\epsilon|s, \mathcal{P})$ is the PDF of the FGM part. Clearly, for $q = 1$, $P_{\text{FGM}}(\epsilon|s, \mathcal{P}) = P_\lambda(\epsilon)$ with λ calculated from equation (7).

For general values of q, the effect of pseudo epistasis should be carefully addressed. Specifically, in the case of sign epistasis, the epistasis is replaced by $-s$ with probability $1-q$. This condition is succinctly encoded by the following equation:

$$P_{\text{FGM}}(\epsilon|s, \mathcal{P}) = (1-q)\delta(s+\epsilon) \int_{-\infty}^{-s} P_\lambda(z)\, dz + q\theta(-s-\epsilon) P_\lambda(\epsilon) + \theta(s+\epsilon) P_\lambda(\epsilon), \tag{24}$$

where $\delta(x)$ and $\theta(x)$ are the Dirac delta function and the Heaviside step function, respectively. This type of construction is in general called survival analysis of left-truncated data [9]. In this expression, the Heaviside step function is

used to combine both types of data points separated by the line $\epsilon = -s$ and the Dirac delta function is introduced to localize the probability at $\epsilon = -s$ when such replacements occur. By adopting the Gaussian approximation for the distribution $P_\lambda(\epsilon)$ of epistasis, $P_{\mathrm{FGM}}(\epsilon|s,\mathcal{P})$ is explicitly written as

$$\begin{aligned} P_{\mathrm{FGM}}(\epsilon|s,\mathcal{P}) =& q \times \frac{1}{2}\mathrm{erfc}\left(\frac{\mathbb{E}_\lambda(\epsilon)+s}{\sqrt{2\left[\mathbb{E}_\lambda(\epsilon^2)-\mathbb{E}_\lambda(\epsilon)^2\right]}}\right)\delta(s+\epsilon) \\ &+(1-q)\frac{\theta(-s-\epsilon)e^{-\frac{(\epsilon-\mathbb{E}_\lambda(m))^2}{2\left[\mathbb{E}_\lambda(\epsilon^2)-\mathbb{E}_\lambda(\epsilon)^2\right]}}}{\sqrt{2\pi\left[\mathbb{E}_\lambda(\epsilon^2)-\mathbb{E}_\lambda(\epsilon)^2\right]}} + \frac{\theta(s+\epsilon)e^{-\frac{(\epsilon-\mathbb{E}_\lambda(\epsilon))^2}{2\left[\mathbb{E}_\lambda(\epsilon^2)-\mathbb{E}_\lambda(\epsilon)^2\right]}}}{\sqrt{2\pi\left[\mathbb{E}_\lambda(\epsilon^2)-\mathbb{E}_\lambda(\epsilon)^2\right]}}, \end{aligned} \tag{25}$$

where the dependencies on the model parameters n, ρ and s_0 implicitly enter via the equations (14) and (15). Finally, the function $\mathcal{L}$ is constructed from the set of all data points $\mathcal{D}$ of the form $\{s, m\}$ as follows:

$$\mathcal{L}(\mathcal{P}) = \sum_{\{s,m\}\in\mathcal{D}} \ln P_{\mathrm{tot}}(m|s,\mathcal{P}). \tag{26}$$

Even though the explicit functional form of $P_{\mathrm{tot}}(m|s,\mathcal{P})$ can be expressed in terms of error functions using (23), it is omitted here as it does not provide further intuition.

Once $\mathcal{L}(\mathcal{P})$ is defined, the remaining task is to find the parameters $(\rho^*, n^*, s_0^*, q^*)$ that minimize $\mathcal{L}(\mathcal{P})$. For each medium, the standard deviations σ_{Error} of the measurement error are given to be $0.04 \times s_m$ for CM and $0.03 \times s_m$ for MM, where the s_m's are defined as the largest single mutant selection coefficients observed in the respective data sets ($s_m = 49$mm and $s_m = 40$mm, respectively). Additionally, the Hessian matrix around the point $(\rho^*, n^*, s_0^*, q^*)$ yields the 95% confidence intervals for these parameters. For our data sets, the estimated parameters were found to be $n^* = 19.3 \pm 4.15$, $\rho^* = 6.89 \pm 0.72$, $s_0^*/s_m = 1.41 \pm 0.25$ and $q^* = 0.21 \pm 0.49$ for CM and $n^* = 34.8 \pm 7.35$, $\rho^* = 9.81 \pm 0.97$, $s_0^*/s_m = 1.62 \pm 0.33$ and $q^* = 1 - 0.90$ for MM. Since the estimated q^* for MM hits the boundary of the valid range $q \in (0, 1)$, the confidence interval is obtained by the profile method rather than from the Hessian matrix. Here, the estimate $s_0^*/s_m = 1.41$ means that the fitness of the phenotypic optimum exceeds the largest observed fitness mutant fitness by a factor 1.41. As expected, we obtained a small value of q^* in CM owing to the large number of data points located near the $-s$-line while we obtained $q^* = 1$ in MM since there are only two data points below $-s$ and one of them is far below the line and hence must be attributed to fast germination. The estimated mean epistasis curves are drawn together with the experimental data in Fig. 3B of the main text.

Furthermore, it is natural to ask whether our estimated FGM parameters also match the experimental single effect size distributions. In Fig. 2B of the main text, we display histograms of the single effect data in comparison to the analytical probability density function (8) evaluated with our estimated parameters. It is shown that they are in a good agreement for $s > 0$. The noticeable deviation in the regime $s < 0$ is not surprising as deleterious mutations were not targeted by the experimental selection assay.

2. *Estimating the frequency of sign epistasis*

As a consistency check, we calculated the distribution of the number of instances of sign epistasis from FGM using the model parameters estimated from the previous analysis. For a given selection coefficient s in the presence of measurement error, the probability of having sign epistasis $P_{\mathrm{sign}}(s)$ is given by

$$P_{\mathrm{sign}}(s,m) = \frac{\int_{-\infty}^{m+s} P_{\mathrm{FGM}}(m-\xi|s,\mathcal{P})P_e(\xi)\,d\xi}{\int_{-\infty}^{\infty} P_{\mathrm{FGM}}(m-\xi|s,\mathcal{P})P_e(\xi)\,d\xi}. \tag{27}$$

In other words, the indicator $X_{s,m}$ of having sign epistasis is a Bernoulli random variable that is unity with probability $P_{\mathrm{sign}}(s,m)$. The total number of instances of sign epistasis N_{sign} is then written as a sum of the indicators, i.e.,

$$N_{\mathrm{sign}} = \sum_{\{s,m\}\in\mathcal{D}} X_{s,m}. \tag{28}$$

The resulting distribution is called a Poisson binomial distribution and its probability densities for both media are shown in Fig. 3C of the main text. The observed number of instances of sign epistasis ($N_{\mathrm{sign}} = 16.2$ for CM and $N_{\mathrm{sign}} = 2.0$ for MM) are shown to be consistent with the distribution. Here, the observed frequencies were obtained by summing all the weights that account for the probability that the measured epistasis originates from sign epistasis.

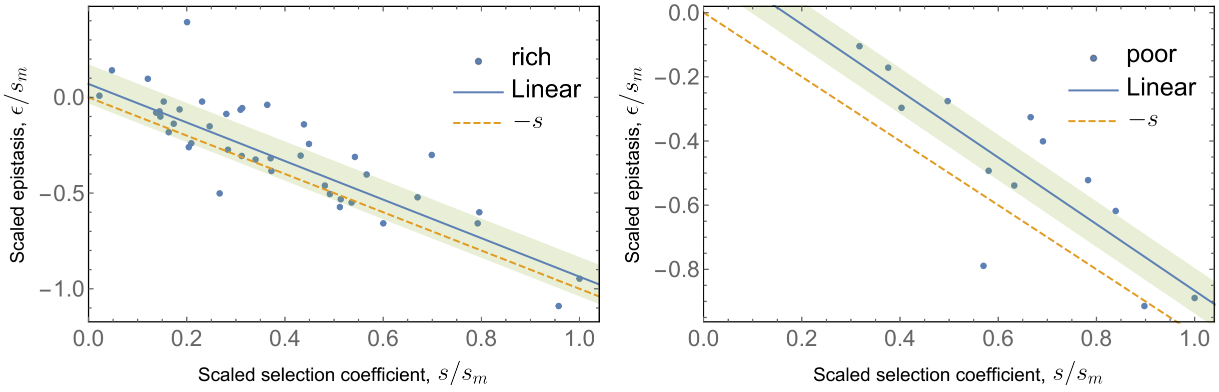

FIG. S3. Epistasis as a function of mean relative selection coefficient for CM (left) and MM (right). The blue line in each panel is the best linear fit predicted from the maximum likelihood method and the shaded region is the 99% variability induced by measurement error.

3. Comparison with a linear model

As a minimal description of diminishing returns epistasis, one can perform a similar analysis using a linear model. Unlike FGM which defines a full distribution of epistasis for any s, the linear model only specifies the mean behavior without inherent variability. As will be shown below, a linear model that accounts for variability only in the form of measurement error, thus assuming that epistasis is a deterministic, linear function of s, cannot explain the large deviation observed in our data. Hence, such an intrinsic variability (with unknown source) is added to the model as an additional model parameter.

To be precise, we assume that the pairwise epistasis satisfies the linear relation $\epsilon(s) = a + bs + \xi$ where ξ is a Gaussian random variable with standard deviation Σ. Following the same procedure as for FGM, the corresponding PDF for the measured epistasis is constructed as

$$P_{\text{Linear}}(m|s,\mathcal{Q}) = q \times \frac{1}{2}\text{erfc}\left(\frac{a+bs+s}{\sqrt{2}\Sigma}\right)\delta(s+m) + (1-q)\frac{\theta(-s-m)e^{-\frac{(m-(a+bs))^2}{2\Sigma^2}}}{\sqrt{2\pi}\Sigma} + \frac{\theta(s+m)e^{-\frac{(m-(a+bs))^2}{2\Sigma^2}}}{\sqrt{2\pi}\Sigma}, \tag{29}$$

where $\mathcal{Q}$ is the set of model parameters, i.e., $\mathcal{Q} = \{a, b, \Sigma, q\}$. Then the full distribution and the log-likelihood function are obtained similarly to (23) and (26). Subsequently, the parameters minimizing the function are determined for both media. The estimated parameters are found to be $(a, b, \Sigma, q) =$ (0.07, -1.00, 0.18, 0.77) for CM and (0.11, -1.04, 0.14, 0) for MM. In both cases the slope of the linear model is close to -1.

In order to select the best model among several possible candidates one generally compares their scores in terms of the Bayesian Information Criterion (BIC) [10]. In our case, the difference in BIC's between the two models, $\Delta\text{BIC} \equiv \text{BIC}_{\text{Linear}} - \text{BIC}_{\text{FGM}}$, is found to be $\Delta\text{BIC} = (-32.47) - (-32.29) = -0.18$ for CM and $\Delta\text{BIC} = (-3.76) - (-3.45) = -0.30$ for MM. Hence, these values suggest that the linear model is favored for both media. However, the relatively small absolute values of ΔBIC ($|\Delta\text{BIC}| < 6$) imply that no clear preference for one of the models can be inferred from this analysis [10].

It is also interesting to check the BIC's for the linear model with $\Sigma = 0$, where we do not allow for any intrinsic variability. Not surprisingly, the BIC's become much worse (392.3 for CM and 270.7 for MM) without changing the estimates of a and b within the significant digits. This shows that the inherent variability is an essential feature of the epistasis data analyzed in this work. As shown in Fig. S3, a large fraction of data points lie outside the shaded 99% variability region, implying that the observed variability cannot be attributed only to the measurement error.

4. Parameter estimation from single effect distribution

An alternative approach to estimating the parameters of FGM would be to fit the predicted distribution (8) to the observed single mutational effects. This approach might seem more natural and facilitates the analysis by removing the issue of survival analysis. However, as shown in Fig. S4, we find that the log-likelihood functions constructed from

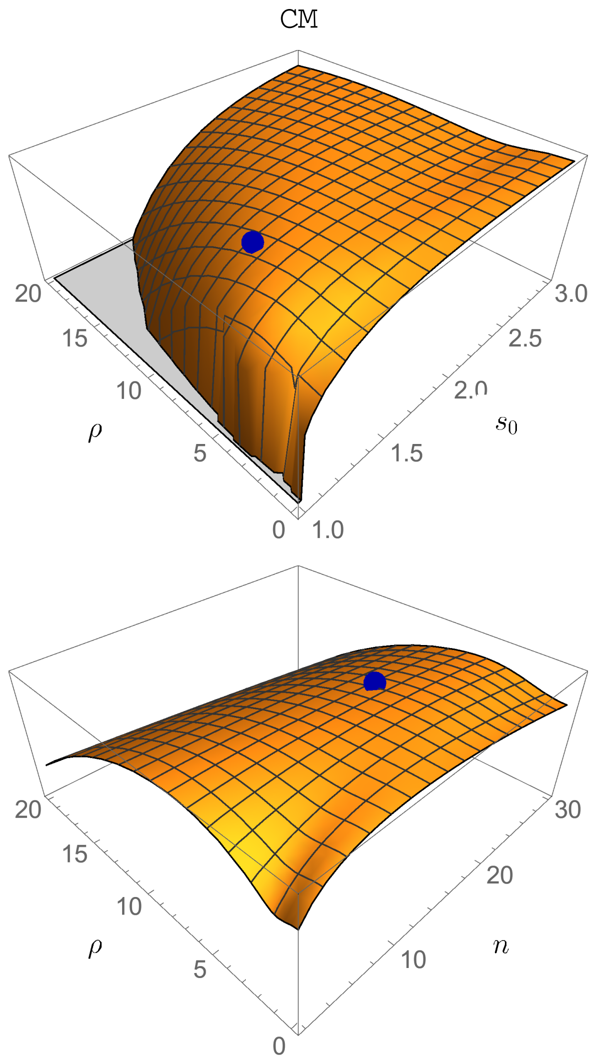

FIG. S4. The log-likelihood of the predicted single effect distribution (8) for CM and MM. The parameters n (upper panels) and s_0 (lower panels) are fixed to the optimal value obtained from the epistasis analysis. It is found that the resulting log-likelihood function forms a plateau in the parameter space. The blue dots represent the optimal point that was estimated by pair-wise epistasis. As expected, they are located inside or at least near the plateau.

(8) for both media are slowly varying in the parameter space of FGM. This results in large confidence intervals for each parameter which in turn makes the inference highly ambiguous. In Fig. 2B of the main text, we added a predicted single effect distribution by using an arbitrary set of parameters inside the plateau to show the resemblance of the curves in the observable interval $s > 0$. Specifically, we used $n = 19, \rho = 20, s_0 = 3$ for CM and $n = 34, \rho = 20, s_0 = 3$ for MM. In the figure, both curves predict the histogram rather well in the beneficial regime $s > 0$ but differ strongly for $s < 0$.

5. *A correction to the Pearson correlation coefficient due to measurement error*

In [11], the authors pointed out that observations of diminishing returns epistasis in terms of the Pearson correlation coefficient between epistasis and single effect size can be biased due to measurement error. Following the approach outlined in [11], we corrected for the measurement error in our data and found a rather strong negative correlation that even becomes more pronounced after the correction, decreasing from -0.849844 to -0.867583 for CM and from -0.826546 to -0.836044 for MM. It can be seen from Fig. 1 of [11] that the correction indeed enhances the negative correlation for sufficiently negative values of the correlation coefficient. From this analysis, we can conclude that the measurement error in our data is rather weak compared to the trend of diminishing returns.

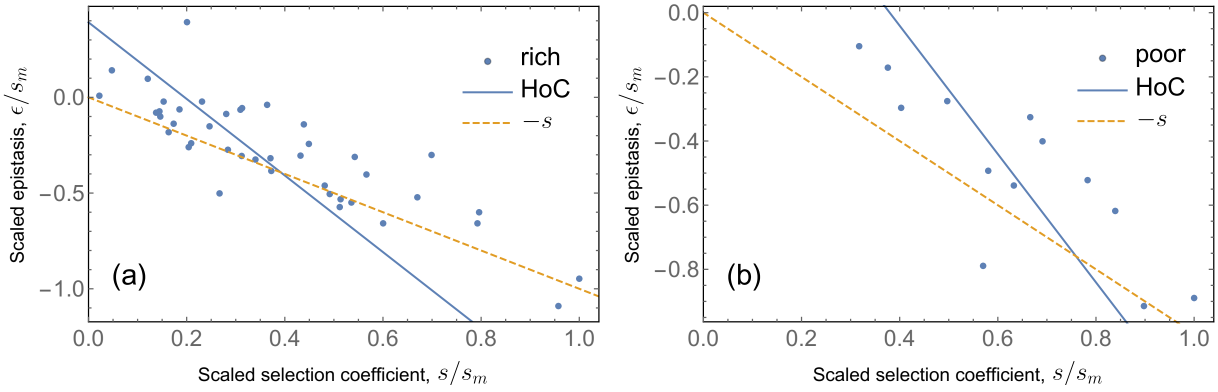

FIG. S5. The epistasis curves (solid) with the estimated parameters for the HoC model. Both curves fail to capture the overall trend of the data. The auxiliary curve $\epsilon = -s$ (dashed) is drawn to show the region where sign epistasis occurs.

II. HOUSE OF CARDS MODEL

Another simple statistical model of epistasis is the House-of-Cards (HoC) or Mutational Landscape model, where the fitness values of different genotypes are independent random variables drawn from a common probability distribution [12–14]. In this model a negative correlation between epistasis and selection strength arises from a rather trivial mechanism, which is an extreme case of the effect of regression to the mean described in [11]. Indeed, if the fitness of the double mutant is a random variable uncorrelated with the single mutant selection coefficients and the latter are conditioned to be equal to s, it follows immediately from the definition (9) that the mean epistasis is

$$\mathbb{E}(\epsilon) = \bar{s} - 2s \tag{30}$$

where $\bar{s}$ denotes the mean selection coefficient.

To provide some perspective on the comparison of the experimental data to the predictions of FGM, we performed a similar data analysis for the HoC model. For the distribution of fitness effect we chose the exponential distribution

$$M(s) = \frac{1}{a} e^{\frac{s_c - s}{a}}, \tag{31}$$

for $s \in (s_c, \infty)$, giving rise to a two-parameter model specified by a and s_c. The mean selection coefficient is $\bar{s} = s_c + a$.

Within the HoC model, the distribution of epistasis conditioned on the two single mutations having the same effect size s reads

$$\begin{aligned} P_{\text{HoC}}(m|s, \mathcal{H}) =& q \times \left(1 - e^{\frac{s_c - s}{a}}\right) \delta(s+m) \\ &+ (1-q) \frac{e^{\frac{-m-2s+s_c}{a}} \theta(m+2s-s_c)}{a} \theta(-s-m) + \frac{e^{\frac{-m-2s+s_c}{a}}}{a} \theta(s+m), \end{aligned} \tag{32}$$

where $\mathcal{H} = \{a, s_c, q\}$. From this expression, the log-likelihood function is constructed analogously to (23) and (26). By minimizing $\mathcal{L}_{\text{HoC}}(\mathcal{H})$, the three parameters (a, s_c, q) were determined as $a^* = 0.37$, $s_c^* = 0.02$ and $q = 0.89$ for CM and $a^* = 0.41$, $s_c^* = 0.35$ and $q = 0.47$ for MM. In contrast to FGM, the epistasis curves with the estimated parameters are not in good agreement with the data, see Fig. S5. Specifically, the BIC values for the two models are -32.29 (FGM), -7.30 (HoC) for CM and -3.45 (FGM), 11.28 (HoC) for MM. For both media, the BIC suggests that FGM is a much better model than HoC.

---

[1] H. A. Orr, Nat Rev Genet **6**, 119 (2005).
[2] O. Tenaillon, Annual Review of Ecology, Evolution, and Systematics **45**, 179 (2014).
[3] G. Martin, Genetics **197**, 237 (2014).
[4] G. Martin, S. F. Elena, and T. Lenormand, Nat Genet **39**, 555 (2007), 10.1038/ng1998.
[5] F. Blanquart, G. Achaz, T. Bataillon, and O. Tenaillon, Evolution **68**, 3537 (2014).
[6] Even though (14) and (15) were obtained under the assumption $n \geq 2$, the expressions remain valid for $n = 1$.

[7] D. M. Weinreich, R. A. Watson, and L. Chao, Evolution **59**, 1165 (2005).
[8] F. J. Poelwijk, D. J. Kiviet, D. M. Weinreich, and S. J. Tans, Nature **445**, 383 (2007).
[9] J. Klein, *Survival analysis : techniques for censored and truncated data* (Springer, New York, 2003).
[10] R. E. Kass and A. E. Raftery, Journal of the American Statistical Association **90**, 773 (1995).
[11] D. Berger and E. Postma, Genetics **198**, 1417 (2014).
[12] J. H. Gillespie, Evolution **38**, 1116 (1984).
[13] H. A. Orr, Evolution **56**, 1317 (2002).
[14] J. A. G. de Visser and J. Krug, Nat Rev Genet **15**, 480 (2014).
